# Intraclonal Protein Expression Heterogeneity in Recombinant CHO Cells

**DOI:** 10.1371/journal.pone.0008432

**Published:** 2009-12-23

**Authors:** Warren Pilbrough, Trent P. Munro, Peter Gray

**Affiliations:** 1 Australian Institute for Bioengineering and Nanotechnology (AIBN), The University of Queensland, Brisbane, Queensland, Australia; 2 ACYTE Biotech Pty Ltd, Brisbane, Queensland, Australia; University of Edinburgh, United Kingdom

## Abstract

Therapeutic glycoproteins have played a major role in the commercial success of biotechnology in the post-genomic era. But isolating recombinant mammalian cell lines for large-scale production remains costly and time-consuming, due to substantial variation and unpredictable stability of expression amongst transfected cells, requiring extensive clone screening to identify suitable high producers. Streamlining this process is of considerable interest to industry yet the underlying phenomena are still not well understood. Here we examine an antibody-expressing Chinese hamster ovary (CHO) clone at single-cell resolution using flow cytometry and vectors, which couple light and heavy chain transcription to fluorescent markers. Expression variation has traditionally been attributed to genetic heterogeneity arising from random genomic integration of vector DNA. It follows that single cell cloning should yield a homogeneous cell population. We show, in fact, that expression in a clone can be surprisingly heterogeneous (standard deviation 50 to 70% of the mean), approaching the level of variation in mixed transfectant pools, and each antibody chain varies in tandem. Phenotypic variation is fully developed within just 18 days of cloning, yet is not entirely explained by measurement noise, cell size, or the cell cycle. By monitoring the dynamic response of subpopulations and subclones, we show that cells also undergo slow stochastic fluctuations in expression (half-life 2 to 11 generations). Non-genetic diversity may therefore play a greater role in clonal variation than previously thought. This also has unexpected implications for expression stability. Stochastic gene expression noise and selection bias lead to perturbations from steady state at the time of cloning. The resulting transient response as clones reestablish their expression distribution is not ordinarily accounted for but can contribute to declines in median expression over timescales of up to 50 days. Noise minimization may therefore be a novel strategy to reduce apparent expression instability and simplify cell line selection.

## Introduction

Protein biologics are an important and growing segment of the drug industry with over US$80 billion in sales worldwide. Many protein biologics, including monoclonal antibodies, are large, structurally-complex glycoproteins requiring functional human-like post-translational modifications for their *in vivo* activity [1]. Cultured mammalian cells, and particularly Chinese hamster ovary (CHO) cells [2], are generally employed as production hosts because simpler prokaryotic and eukaryotic expression systems lack suitable native glycosylation machinery and may not fold and secrete these biomolecules efficiently [3]. Yet despite their widespread use and commercial significance, two major issues remain unresolved in establishing productive mammalian cell lines, namely clonal heterogeneity [4] and expression instability [5].

Large-scale production of recombinant proteins relies on stable integration of expression vectors into the host genome [6]. Ordinarily this involves non-targeted DNA delivery and chemical selection to integrate and amplify transgene sequences encoding the product [7]. The resulting transfectants differ markedly in expression due to an inherent lack of control over gene dosage and chromosomal context of integrating copies [8]–[10]. Random integration and amplification may also disrupt or dysregulate endogenous genes [11]–[13] creating the potential for variation in other cell traits [14], [15]. Accordingly, production cell lines are ‘cloned’, or derived from a single cell, in order to minimize heterogeneity (International Conference on Harmonisation (ICH), Guideline Q5D, 1997).

Upstream of the cloning step, however, the marked diversity amongst transfectants makes the process of clone isolation a considerable challenge. High producers are rare and those also satisfying product quality and other selection criteria, such as rapid growth, are rarer still [6]. Extensive empirical screening of large numbers of candidate clones is therefore required, which is resource intensive and frequently rate limiting in early development. Protein expression stability also tends to be problematic. Most clones suffer a decline in productivity during the extended culture periods required to reach manufacturing scale, yet this is unpredictable and varies from clone to clone. Efforts to define the molecular determinants of stability [16] have so far achieved only limited success and stability is still routinely assessed by directly monitoring each clone over several months of growth.

Prior examination of these issues has focused chiefly on differences *between* clones isolated from mixed populations, such as those arising from transfection or gene amplification [4], [17]–[24]. We take an alternate approach, exploring the degree of variation *within* a clone, using single cell analysis facilitated by IRES-driven coexpression of intracellular fluorescent markers. Clones are normally assumed to be homogeneous but emerging fundamental research in bacteria [25]–[28], yeast [29]–[34], and more recently mammalian cells [35]–[39], has revealed that gene expression can vary significantly between genetically-identical cells, even in a common environment (reviewed in [40], [41]). We reasoned that this ‘hidden’ source of variation within clones [42], [43] might also have practical implications for cell line development, which are not yet widely appreciated. Indeed, we show in this study that intraclonal heterogeneity contributes materially to clonal variation and even to the apparent instability of expression over time. This fresh perspective may open up new avenues for understanding and overcoming these longstanding problems.

## Results

### Distribution of Expression Levels in a Clonal Population

**Figure 1:**
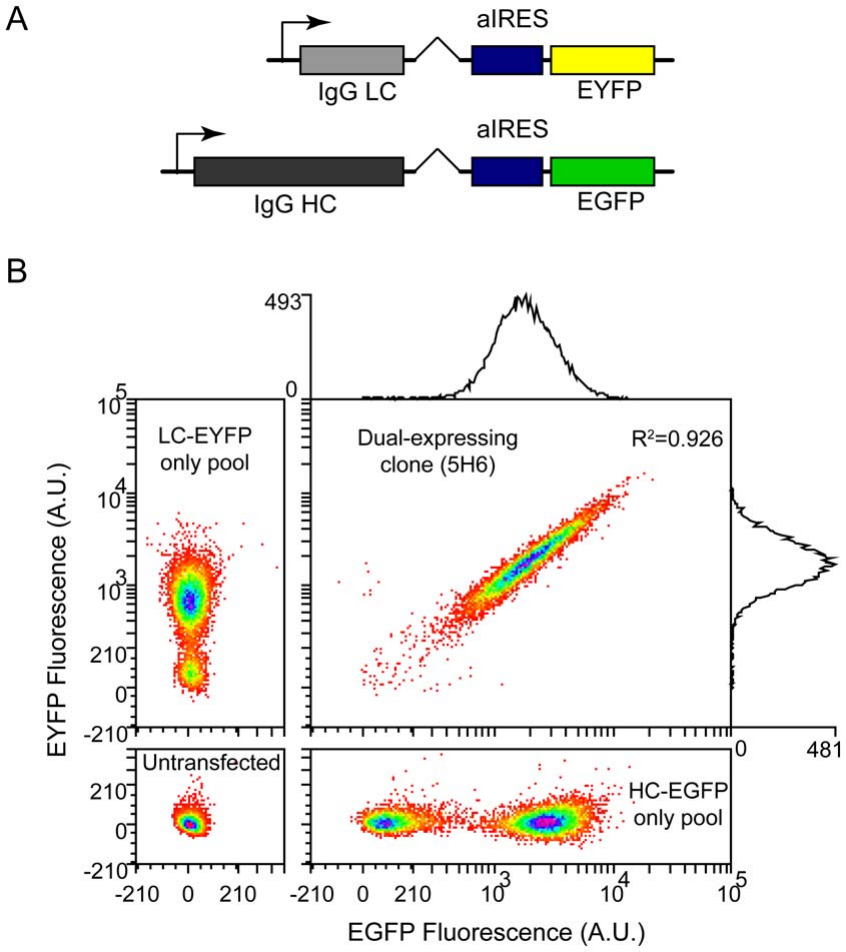
Expression heterogeneity in a clone. A) Bicistronic antibody expression constructs [44] designed to screen IgG4 kappa light chain (LC) and gamma heavy chain (HC) transcription using fluorescent reporter proteins (EGFP and EYFP) translated from the same mRNA by an attenuated encephalomyocarditis virus (EMCV) internal ribosomal entry site (aIRES). A metal-responsive promoter drives transcription (Methods). A hybrid synthetic intron situated immediately upstream of the aIRES improves efficiency of 3′ pre-mRNA processing [95]. Features other than fluorescent proteins and immunoglobulin chains are identical between constructs. B) Bivariate distribution of reporter protein fluorescence in cells from a dual-expressing clone (5H6, cell-specific antibody secretion rate ∼2 pg/cell-day) measured by flow cytometry (main panel). Note split linear-log axes. Spectral overlap and autofluorescence were compensated using single-color controls and untransfected cells (adjacent panels, see Methods). Histogram counts on each axis in main panel are univariate distributions of EGFP and EYFP fluorescence in the clone, with coefficients of variation (CV = s.d./mean) of 0.7. *R* ^2^ in main panel is for linear fit to double-positive cells (fit not shown). 10,000 events shown in each panel. Fluorescence in arbitrary units (A.U.).

We utilized a pair of expression vectors developed for accelerated screening of monoclonal antibody producing cell lines by fluorescence-activated cell sorting (FACS) [44]. Each construct encodes a human immunoglobulin G subclass 4 (IgG4) kappa light chain or gamma heavy chain coupled to enhanced green or yellow fluorescent protein (EGFP or EYFP) by an attenuated internal ribosomal entry site (aIRES) (**Fig. 1A**). EGFP and EYFP serve as chain-specific reporters transcribed from the same promoter and translated proportionally but at a lower rate than the antibody chains [45]. When co-expressed, we found intracellular reporter fluorescence to be correlated with cell-specific antibody secretion at the population level [44], and with ‘cold capture’ cell surface antibody [46] at the single-cell level (**Fig. S1**).

A representative dual-expressing clone (5H6) was isolated from a co-transfected gene-amplified CHO-K1 pool by FACS single cell deposition. This clone, consistent with others we have isolated [44], exhibited considerable cell-to-cell variation by flow cytometry (**Fig. 1B**), despite originating from a single cell. This variation is not apparent in more traditional bulk assays such as ELISA which measure only the population mean. The EGFP and EYFP fluorescence distributions in the clone were unimodal and qualitatively log-normal with coefficients of variation (CV = standard deviation/mean) of between 0.5 and 0.7 (depending on the day of measurement), and signals spanning at least an order of magnitude (**Fig. 1B, main panel**). Uniform calibration beads at a similar fluorescence intensity had a CV of ∼0.05, indicating ample measurement resolution (not shown). Polyclonal gene-amplified single color pools (**Fig. 1B, adjacent panels**) (and co-transfected pools, not shown) were bimodally distributed in the respective channels (possessing both expressing and non-expressing subpopulations). Surprisingly, they otherwise spanned a similar range of fluorescence intensities to the clone, in spite of greater genetic heterogeneity. This suggests expression variation in a clone can be large relative to variation between clones.

Expression levels of the two transgenes were also highly correlated when expressed together in the clone (*R* ^2^ = 0.926), with points lying mainly along the diagonal in a bivariate plot (**Fig. 1B, main panel**). The majority of expression noise therefore exerts an equal influence on both transgenes, maintaining a similar ratio of light to heavy chain transcription despite considerable cell-to-cell variation in the expression of each chain. This was also evident in other dual-expressing clones [44], and may be needed for efficient antibody assembly and secretion [47]. The correlation likely arises from tandem integration of the expression cassettes at a common genomic locus, an outcome favoured by co-transfection [48]–[51]. Genomic proximity is known to enhance coordinated expression [52], [53], which is mediated, for example, by local chromatin folding. The vectors also share common regulatory sequences such as transcription factor binding sites and untranslated regions, which may likewise play a role in coordinated expression, though others have shown that noise correlations are greatly reduced in pairs of otherwise identical expression cassettes integrated at discrete sites [30], [37], [38], suggesting genomic proximity may be the more important factor.

### Contribution of Cell Size and Cell Cycle to Expression Level Variation

**Figure 2:**
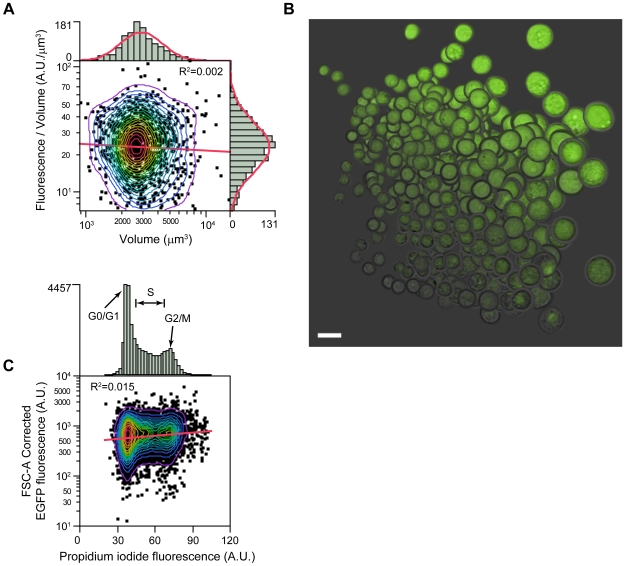
Variation of expression with cell size and cell cycle. A) Fluorescence and cell volume measurements on live cells (Clone 5H6) by imaging flow cytometry. Cell volume inferred by calculation from brightfield projected area (Methods). EGFP and EYFP fluorescence collected in a single channel. Linear fit (red line) and *R* ^2^ are shown in main panel. Contours are percentiles (5%). Histogram counts on each axis are shown fitted to a log-normal distribution (red curves). Fluorescence in arbitrary units (A.U.). B) Cell image field (montage) illustrating subset of events from (A) with image centers aligned to match corresponding graph coordinates. False-color overlay of bright field and fluorescence channels. Scale bar, 20 µm. Note: brightness is perceived per unit projected area, not per unit volume as plotted, making larger cells appear brighter in cross-section due to depth of field. C) Fixed cells (Clone 5H6) stained with propidium iodide for DNA content and measured by conventional flow cytometry. EGFP fluorescence corrected for cell volume (estimated by FSC-A, see Methods). EYFP similar (not shown). Linear fit (red line) and *R* ^2^ are shown in main panel. Contours are percentiles (5%). Histogram counts on upper axis indicate cell cycle phases (G0/G1, S, G2/M). Fluorescence in arbitrary units (A.U.).

To characterize expression level variation in the clone we first sought to examine the influence of non-uniform population structure (cell size and cell cycle phase) that exists during asynchronous growth (**Fig. 2**). By imaging flow cytometry, both cell volume and fluorescence per unit volume (fluorescence concentration) were approximately log-normally distributed and varied respectively over a ∼10-fold range (**Fig. 2A**). This substantial cell-to-cell heterogeneity is well illustrated in the images captured during analysis, which also show that fluorescence is uniformly dispersed within most cells (**Fig. 2B**). While *total* fluorescence did depend in part on cell volume (not shown), fluorescence *concentration* was independent of cell volume (*R* ^2^ = 0.002) (**Fig. 2A**). Thus expression on a volume-corrected basis was not, on average, biased to large or small cells. Furthermore, substantial variation was still present in volume-corrected fluorescence at all cell sizes suggesting factors other than cell size are involved.

Along with cell size, the rate of gene expression could vary during the cell cycle. We found, however, that position in the cell cycle, as measured by DNA content in fixed cells, explained little (<2%) of the variation in reporter fluorescence, once cell size had been accounted for (estimated by forward scatter area, FSC-A) (**Fig. 2C**). This was also true in live cells and when cell size was estimated by imaging flow cytometry (not shown). A lack of cell cycle dependency after correcting for cell size is consistent with at least one prior report [54]. We note that the half lives of EGFP and EYFP are in the order of 24 h [55], limiting their responsiveness to fluctuations with timescales of less than one cell generation (∼15 h). But such dampening of high frequency noise is desirable for screening purposes, and irrespective of whether additional underlying cell cycle fluctuations exist, the variations we do detect are largely independent of the cell cycle.

### Measurement Noise and Dynamic Response of Sorted Subpopulations

**Figure 3:**
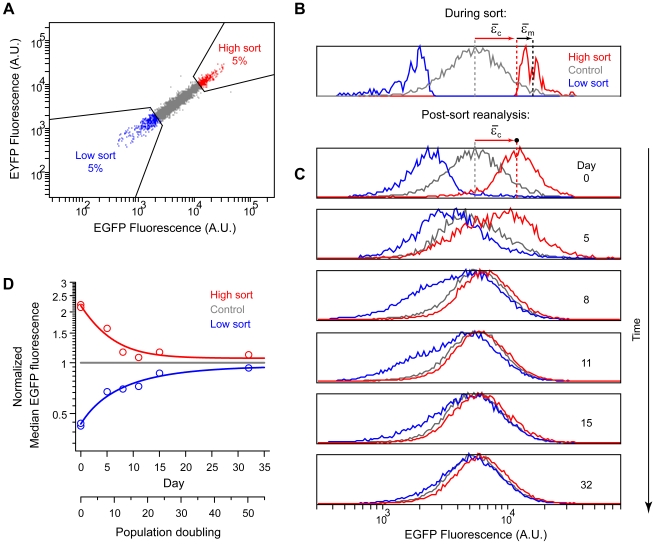
Measurement noise and dynamic response of sorted subpopulations. A) High (red) and low (blue) subpopulations (top and bottom 5% of expressing cells, respectively) were sorted from the clone (5H6) by FACS along with a control population (gray, all expressing cells, including high and low). Fluorescence in arbitrary units (A.U.). B,C) EGFP fluorescence distributions of sorted subpopulations were monitored over time (in the presence of selection). Average deviations due to cell variation (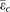) and measurement noise (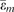) in the high subpopulation are indicated by red and black arrows, respectively. Measurement noise was estimated to be ∼30% of total variation (Methods). The low subpopulation and EYFP channel yielded similar estimates (not shown). Data is from two independent sorting runs. Fluorescence in arbitrary units (A.U.). **D**) Median fluorescence for each subpopulation was normalized to the control and plotted as a function of time (open circles). Relaxation half times (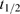) were estimated by fitting a first order exponential decay (lines). 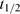 (high sort) = 3 days (∼5 generations); 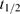 (low sort) = 7 days (∼11 generations). EYFP similar (not shown).

To estimate the relative contribution of measurement noise to observed variation and to establish a timescale for expression fluctuations we tested the dynamic response of subpopulations isolated from the clone. The highest and lowest 5% of expressing cells in the clone were sorted by FACS, along with a control (**Fig. 3A, B**), and reanalyzed at several time points (**Fig. 3C, D**). During the sort, the high and low sort gates represent the truncated tails of the expression distribution in the clone (**Fig. 3B**). Both measurement noise and cellular variation contribute to this distribution. After sorting, we immediately reanalyzed samples of the sorted cells. The distributions of the sorted subpopulations became broader and shifted towards the mean of the control (compared to the original sort gates), though they remained distinctly separated (**Fig. 3C, Day 0**). The relative magnitude of this shift corresponds to the percent measurement noise, in this case ∼30% of total variation (see Methods for details). By comparison, variation in uniform calibration beads was only about 1% of the variation in the clone (CV^2^(beads)/CV^2^(5H6) ≈0.05^2^/0.5^2^ = 1%). This suggests most measurement noise is associated specifically with cell measurements, and probably reflects the non-uniform shape, internal structure, and orientation of cells in the sample stream during flow analysis. Furthermore, although measurement noise is significant, the majority of variation (∼70%) is of biological rather than technical origin, confirming that intraclonal heterogeneity is not simply a measurement artifact.

The persistence of cellular variation was determined by reculturing the sorted subpopulations in the presence of selection. Over about 30 days, the original distribution was progressively reestablished in both high and low subpopulations (**Fig. 3C, Day 5–32**). Thus, cellular variation in the clone is predominantly non-heritable and reverts to a characteristic steady state. Cell size was not a major contributing factor as median FSC-A showed no trend with time and varied little across all sorted populations and time points (mean square error 3%, not shown). The dynamics of relaxation to steady state (**Fig. 3D**) were slow relative to the population doubling time (∼15 h), consistent with prior reports in eukaryotes [39], [56]–[58]. This ‘metastability’ seems to rule out simple growth and division mechanisms, and suggests a degree of mitotic inheritance or ‘cellular memory’. A longer timescale of noise fluctuations (more technically, a longer autocorrelation) can lead directly to a higher noise magnitude [59], which may explain the occurrence of both a relatively long ‘mixing time’ and a high CV in our system, compared to those observed by Sigal and co-workers in human lung carcinoma cells [39]. Interestingly, we found that the dynamic response to sort perturbations was asymmetric, relaxing more quickly from high expression levels (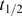 = ∼5 generations) than from low expression levels (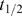 = ∼11 generations) (**Fig. 3D**). This corresponds to mean transmitotic (mother-daughter) correlations of 0.87 and 0.94, respectively [60]. The reason for asymmetric relaxation rates is not well understood but has also been reported elsewhere [36], [56] and is reminiscent of negative feedback. Population dynamics could contribute to asymmetry if growth rates are retarded at high expression levels [61], but cannot be the sole factor driving relaxation as the low sort eventually returns to the same steady state as the high sort, albeit more slowly.

### Dynamic Response of High-Expressing Subclones

**Figure 4:**
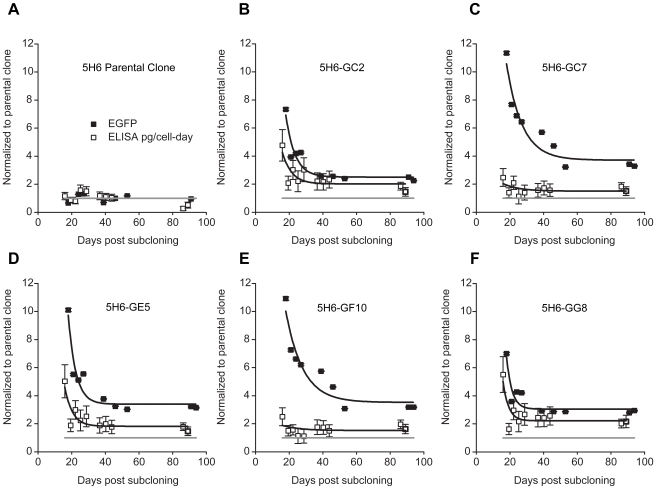
Dynamic response of high-expressing subclones. Five high-expressing subclones were isolated from parental clone 5H6 by stringent FACS sorting. First, the top fluorescing 0.05% of the double-positive population was sorted and recultured for 10 days. Then the top 0.05% of this enriched subpopulation was cloned by single cell deposition into a 96-well plate and the brightest five clonal colonies (5/26) were selected (overall stringency roughly 1 in 20,000,000). A control 96-well plate sorted from the center of the double-positive population yielded 50 clonal colonies, but none of comparable brightness to the selected subclones. Expression dynamics in the parental clone, A) and the five chosen subclones, B–F), were monitored during long-term culture (in the presence of selection) by flow cytometry (median EGFP fluorescence, closed symbols) and ELISA (cell specific antibody secretion rate, pg/cell-day, open symbols). Data is presented in terms of double positive cells, normalized to the parental clone, and fitted to a first order exponential decay (lines). See Methods for details. Horizontal gray lines indicate the level of the parental clone (normalized expression = 1). Error bars are standard errors. 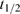 for subclones 5H6-GC2, -GC7, -GE5, -GF10, and -GG8 were 4 days (∼4 generations), 6 days (∼7 generations), 3 days (∼4 generations), 7 days (∼7 generations) and 2 days (∼2 generations), respectively. EYFP similar (not shown).

**Figure 5:**
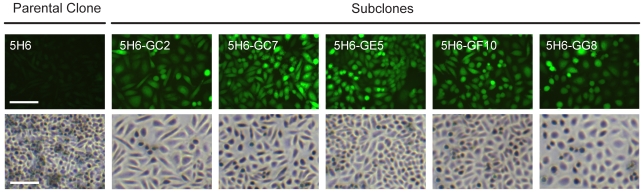
In-situ fluorescence and morphology of sorted subclones. Fluorescence and phase contrast images of the parental clone (5H6) and subclones (5H6-GC2, -GC7, -GE5, -GF10, -GG8) in adherent culture 24 days after subcloning. Scale bars, 100 µm.

Although the bulk of variation in the clone was non-heritable, we sought to find out whether rare spontaneous variants possessing heritable increases in expression could be isolated. We reasoned this would require exceptionally high selection stringency given the extent of background phenotypic variation and the presumed genetic uniformity of the clone. By twice sorting the top 0.05% of the population we obtained five subclones (including possible siblings) with considerably higher fluorescence than the parental clone, 5H6 (**Fig. 4**, **5**). The final sort threshold was approximately 13-fold above the median fluorescence of the parental clone.

We then monitored the dynamic response of the subclones and the parental clone during long-term culture in the presence of selection. At the first timepoint (18 days after isolation), the median EGFP fluorescence of the subclones was still approximately 7- to 11-fold higher than the parental clone (**Fig. 4B–F**), a greater perturbation than the earlier subpopulation sort (**Fig. 3**) due to the higher sort stringency. Variation within each of the subclones (CV = 0.5 to 0.7) was already comparable to the parental clone (CV = 0.5 at this timepoint) and did not trend with time or expression level thereafter (not shown). This suggests cells undergo rapid phenotypic diversification but variation is constrained and reaches steady state.

Over subsequent time points, median EGFP fluorescence decayed by several fold. The kinetics were similar to the previous subpopulation sort (**Fig. 3**) (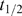 = ∼2–7 generations) suggesting a common mechanism. Overall it took about 30–50 days for expression levels in the subclones to stabilize (**Fig. 4B–F**). The declines were similar in both magnitude and duration to those normally seen during recombinant cell line development [4], [23], [24], [51], yet no underlying instability was observed in the fluorescence of the parental clone, which remained constant over the same time period (**Fig. 4A**). This is evidence that displacement from steady state can contribute to a perceived lack of stability, something not previously considered.

The steady state fluorescence reached in the subclones was about 2.5- to 4-fold above that in the parental clone, implying that the subclones did indeed possess heritable increases in expression. Notably, however, the transient decay in fluorescence was larger than the eventual differences in steady states, again underscoring the relative dominance of non-heritable variation.

We used secretion assays at the population, colony, and single cell level to confirm higher plateau expression in the subclones (**Fig. S2**). Specific antibody secretion rates (pg/cell-day) measured by ELISA obeyed similar kinetics to intracellular fluorescence, but the relative increases over the parental clone were smaller, particularly in subclones 5H6-GC7 and 5H6-GF10, whose secretion rates changed only slightly despite large changes in reporter fluorescence (**Fig. 4C, E**). This suggests transcriptional increases are not fully passed on to secretion, consistent with several prior reports [51], [62]–[64]. In circumstances where only ELISA measurements are performed, these subclones would appear stable [16]. Additionally, our results demonstrate that the severity of the secretion bottleneck can vary markedly, even between closely-related subclones.

Subclones were also assessed for mean cell size, DNA index, and doubling time. All subclones were DNA hyperdiploid with a larger mean cell size than the parental clone, though cell volume alone was not sufficient to account for expression increases (**Table S1** and **Fig. 4**). On the other hand, larger mean cell size did coincide with longer doubling times (**Fig. S3**) and altered culture morphology (**Fig. 5**). Apparent doubling times also increased during periods of higher expression in each subclone, but the relationship was not as clear between subclones (**Fig. S4**). Furthermore, although cells of similar fluorescence intensity tended to be spatially clustered in culture (**Fig. 5**), no systematic link to flask location or confluence was evident to implicate cell microenvironment as the principal cause of expression heterogeneity. Instead, such clustering probably arose from the partial mitotic inheritance of expression levels, as already described. Lastly, the effect of intracellular pH variation and IRES-specific regulation on reporter fluorescence are also possible considerations (**Text S1**).

## Discussion

When establishing stable cell lines, considerable variation is observed between clones, which has traditionally been attributed to genetic heterogeneity in the transfectant pools from which the clones are isolated. We show that in addition to genetic heterogeneity, a significant fraction of total variation may arise from phenotypic differences between cells in each pure clone making up a pool. This, in turn, appears to result from random expression fluctuations in individual cells over time, as elegantly demonstrated in the landmark study of Sigal *et al*. [39]. Since phenotypic variation is ultimately non-heritable, the exploitable diversity in transfectant pools may be less than previously thought. The combination of novel intracellular transcription markers and high-throughput single-cell analysis, along with the simplicity and sensitivity of our method, was crucial to this advance.

Our results also raise the intriguing possibility that intraclonal expression noise and positive selection bias may together contribute to the apparent instability of freshly isolated clones. Stable high producers are rare, and in the presence of sufficient intraclonal variation, may be obscured by the upper tails of the more abundant low producer population. These upper tails represent the small fraction of low producers temporarily in a high expression state due to expression variation. A stringent selection threshold improves the likelihood of isolating true high producers over background variation, but also more strongly biases for cells perturbed above steady state at the time of selection. As we have shown, if non-heritable variation is dominant, the downward mean reversion may be more substantial than the ultimate increase in steady state expression arising from stringent selection. Selection bias may therefore be partly responsible for the widespread belief that high producers are more unstable than low producers [5]. We show that expression can equally increase with time if the selection bias is negative, which may not have been realized previously as low producers are rarely the target of screening. This dissipation of non-heritable variation may thus explain some anecdotal reports of clone rankings changing from cloning through scale-up [65], [66]. Rather than being unstable, these clones may simply have not reached steady state. The relative importance of this mechanism compared with more well-established modes of expression instability [5], such as the permanent loss or rearrangement of transgene copies, will depend on the amplitude and persistence of expression fluctuations, which are expected to be system-dependent. Thus, whether transient effects are generally as significant as seen in this study remains to be determined. We have, however, made similar observations with other promoters such as the murine cytomegalovirus (mCMV) promoter, suggesting this degree of variation is not strictly promoter specific (unpublished data).

Expression noise is not limited to transgenes [38], [39], [57], [58], [67] or transformed cells [36], [68]–[74]. Indeed, it is becoming increasingly evident that a wide repertoire of endogenous genes fluctuate stochastically [29], [36], [72], [73], [75], [76], particularly in culture [77], resulting in an enormous diversity of physiological states, which may also have an indirect effect on transgene expression. Non-genetic variation enables cells to adapt to short-lived environmental changes without permanently accumulating potentially harmful mutations [31], [78]. Phenotypes must persist long enough for survival of daughter cells but not so long that diversity cannot be regenerated quickly. This intermediate timescale is consistent with our present observations. For highly expressed proteins in eukaryotes, stochasticity arises primarily from random bursts of promoter activation [26], [27], [38], [79]–[81] amplified by transcription and translation [28], [32], [34], and turnover of mRNA and protein [38], [82]. Promoter activation seems to coincide with movement of free chromatin loops in and out of ‘transcription factories’ [83]. Indeed, the presence of transcriptional bursting in CHO cells was recently verified by Raj *et al*. [38], who employed fluorescence in-situ hybridization to directly visualize single mRNA molecules produced from an integrated reporter gene. Bursting, however, typically involves timescales of minutes in prokaryotes and lower eukaryotes [26], [27], [79] and hours in mammalian cells [38]. Furthermore, proteins are degraded and diluted by growth, with a maximum half-life of about a cell generation. In order to achieve the longer time constants we have observed, transitions must be slower than this, perhaps in the order of days. Simply having less frequent promoter bursts at a fixed low rate is expected to yield expression distributions where many cells do not contain detectable levels of the protein of interest [38], [81], [82]. Instead, to give a realistic representation of our results, the rate constants themselves must presumably undergo slow changes via additional layers of extrinsic regulation [39], perhaps involving random low frequency modulation of the underlying burst size or frequency. As others have suggested, chromatin is one logical candidate to mediate such regulation [30], [32], [38]. In fact, random fluctuations in chromatin folding have previously been linked to fluctuations in expression [84], and chromatin inheritance timescales are consistent with those prevailing in our system [85]. It is also possible, though we believe less likely, that gains and losses of transgene copies or other factors such as genomic instability may be involved in the dynamic and reversible shifts in expression level we have observed. Moreover, although we have focused here on graded fluctuations, binary switching between expressing and non-expressing states are also possible [37], [84]. The putative connection with chromatin raises the fascinating prospect of a functional link between expression noise and epigenetic gene silencing.

Predicting expression stability would be an important means to accelerate cell line development. The fact that intraclonal variation has not been widely recognized, and is apparently probabilistic in nature, may explain why this has been difficult to achieve. The precise molecular basis for random expression fluctuations remains a matter for future research, but even if epigenetic markers become available to predict stability, molecular characterization of every clone is still likely to be too unwieldy for routine screening. A simple alternative is to profile expression levels in early clonal populations by flow cytometry to identify those which are most homogeneous [50], [66]. A justification for this approach is now clear in light of our results— clones with tight distributions are more likely to be at or near steady state and to experience only small transient adjustments in their median expression level. Extending this principle, we predict that to improve consistency and apparent stability across all clones, a novel strategy would be to engineer reduced intraclonal expression noise into the vector-host system. Low noise promoters [31], [32], [76], noise-suppressing endogenous genes [32], [86], favorable integration sites [30], [87], and recruitment of chromatin-opening or barrier elements [88]–[90] are potentially promising avenues. Mechanistically, the most effective way to lower noise (CV) whilst maintaining or increasing expression, is to reduce the ‘burstiness’ of promoter activation, either by increasing the switching rate of promoter states, increasing the fractional promoter ‘on’ time, or minimizing pauses in elongation [29], [32], [40], [91], [92]. Alternatively, bursts may be smoothed by adding independent or anticorrelated transgene copies [33], [38], [91] in place of fully correlated copies that arise from tandem integration and coamplification. In addition to noise amplitude, we also envisage that noise kinetics could be manipulated to facilitate clone screening, either to achieve steady state more quickly for early comparison of clones, or to extend transient dynamics so their effects are minimized during the culture scale-up period.

## Methods

### Cell Lines and Cell Culture

CHO-K1 cells (ATCC CCL61) and derivative cell lines were maintained in DMEM/F12 medium (Invitrogen) supplemented with 10% fetal bovine serum (FBS) and 5 mM L-glutamine or Glutamax® (L-Ala-L-Gln dipeptide; Invitrogen) in tissue culture treated plates or vent cap flasks in a humidified incubator at 37°C and 5% CO_2_. Cells were subcultured every 3–4 days by rinsing with Dulbecco's phosphate buffered saline (DPBS), detaching with TrypLE™ (Invitrogen), and quenching with complete medium. Cell counts were performed by haemocytometer or Cedex HiRes (Innovatis).

### Expression Vectors, Stable Transfection, Amplification, and Cloning

The antibody expression vectors have been described previously [44]. Briefly, the vector backbone [93] contains a neomycin selection marker, full length human metallothionine II_A_ gene (h*MTII_A_*) as an amplifiable marker, and a metal-hyperinducible promoter M2.6(Δ) derived from h*MTII_A_* to drive transgene expression [94]. See also **Fig. 1A**. The synthetic intron and attenuated IRES originate from the pIRES series of vectors (Clontech). Transfectants were generated by electroporation, selection in 400 µg/ml G418, and amplification in metal up to final concentrations of 100 µM ZnSO_4_ and 6 µM CdCl_2_, as described previously [44]. These G418 and metal concentrations were then maintained continuously, providing sustained selection pressure and promoter induction. Cloning was performed by FACS single cell deposition into 96-well plates. Clonality was assessed immediately after sorting by microscopic observation to identify wells containing only a single cell.

### Flow Cytometry and FACS

Analysis and sorting was performed on a BD FACSAria cell sorter (Becton Dickinson) equipped with an automatic cell deposition unit for sorting into plates. Cells were prepared by mixing and straining through a 70 µm nylon mesh prior to analysis or sorting. EGFP and EYFP were excited with a 13–20 mW Coherent® Sapphire™ solid state laser at 488 nm, and emissions collected with HQ510/20 BP and HQ550/30 BP filters (Chroma). At least 10,000 events, and routinely 50,000 events, were acquired for each sample. Debris and doublets were excluded by gating on forward scatter and side scatter dot plots (FSC-A vs. SSC-A, FSC-W vs. FSC-H, SSC-W vs. SSC-H). Dead cells were excluded by propidium iodide (2 µg/ml) and/or FSC and SSC (back-gated from PI). Compensation for spectral overlap and autofluorescence was performed each day using untransfected and single-transfected control cell lines (**Fig. 1B**), and single-stained untransfected cells. Automatic instrument compensation was manually fine-tuned on split linear-log dot plots to match median fluorescence values of negative and positive subpopulations. Special care was taken to achieve precise compensation in order to eliminate artificial correlations. Sphero 8-peak Rainbow calibration beads (Spherotech) were used to set detector voltages each day, standardize fluorescence measurements performed on different days, and establish instrument resolution. All cell sorting was performed with a 100 µm nozzle at 28 psig sheath pressure, using the highest purity sort mask (single cell mode on the FACSAria). Data was acquired with BD FACSDiva™ software (v5.0.1 or v6.0) and further analyzed with WEASEL v2.5 (Walter and Eliza Hall Institute of Medical Research).

### Imaging Flow Cytometry

An Imagestream® 100 imaging flow cytometer (Amnis Corporation) was used to collect data for **Fig. 2A, B**. EGFP and EYFP were excited with the 488 nm laser and fluorescence emissions were collected together in the 500–560 nm channel. Brightfield, darkfield, and fluorescence images were captured for each event. Image analysis was carried out with Amnis IDEAS® software (v3.0), using default preprocessing settings. Fluorescence compensation was performed using the built-in algorithm in best-fit mode, and fine-tuned manually. Cell doublets and clumps were excluded using various shape features calculated from the brightfield images (area, aspect ratio, circularity, compactness, perimeter, and shape ratio). Unfocused cells were gated out using contrast and gradient RMS features in the brightfield channel. PI was used to discriminate dead cells. The final gate, consisting of in-focus, viable, reporter-positive single cells, comprised 1,171 events.

### Cell Volume Estimation and Forward Scatter Correction

Cell volume was estimated from projected area measured by imaging flow cytometry. Specifically, the default brightfield segmentation mask was eroded by 3 pixels to give a closer fit to the cell, and area (A) was determined by summing pixels in the mask (each pixel 0.25 µm^2^). Volume (V) was then calculated from area-equivalent diameter, according to V = 4/3π(A/π)^3/2^. This assumes cells are spherical, leading to slight overestimation of true volume if cells deviate from perfect sphericity. For our needs the estimates were adequate, particularly since projected cell images (**Fig. 2B**) exhibited consistently high circularity. To account for cell size in conventional flow cytometric analyses where images were unavailable, we used forward scatter area (FSC-A) as a surrogate for cell volume (**Fig. 2C**). Fluorescence was detrended from FSC-A by linear regression. Residuals from the fit were added to median fluorescence, effectively removing the correlation with FSC-A.

### Cell Cycle Analysis

Cell cycle analysis was performed on a BD LSR II flow cytometer. Cell preparation steps were performed on ice and designed to maximize retention of intracellular fluorescence. Cells were fixed in 1% formalin, permeabilized in 75% ethanol, and incubated overnight in PBS with 250 µg/ml RNAse and 2.5 µg/ml PI, prior to analysis.

### Measurement Noise Estimation

We used a variance component model to extract measurement noise estimates from fluorescence distributions collected during and immediately after cell sorting. Single cell fluorescence (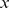) (in either EGFP or EYFP channels) was modeled by 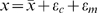, where 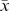 is the ensemble average fluorescence, 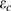 is the deviation in fluorescence of a given cell due to cellular variation, and 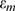 is the deviation in fluorescence of a given cell due to measurement noise (all variables log-transformed). The (log-transformed) deviations were assumed to be normally distributed (consistent with experimental observations), with zero mean and variance proportional to each noise source, i.e. 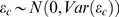 and 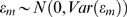. According to this model, the overall mean deviation or bias in the gated subpopulation during sorting is 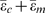, where 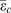 and 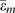 are the average deviations due to cellular variation and measurement noise, respectively (**Fig. 3B**). The same cells reanalyzed immediately after sorting were assumed to be unchanged by the process of sorting and the brief time elapsed, with 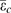 retaining the same bias. Conversely, the new measurement was independent of the first, regenerating a full and unbiased measurement error distribution, with 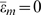 (**Fig. 3C** **, Day 0**). From the two measurements, 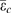 and 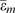 in the sort gate are easily determined: 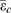 is the average deviation of the sorted cells in the reanalysis, and 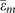 is the difference between the average deviation of sorted cells in the sort gate (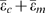) and the reanalysis (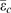). The ratio of measurement bias to total bias in the sort gate (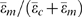) is then equal to the ratio of measurement variance to total variance (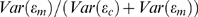), a relationship we verified by Monte-Carlo simulation. This ratio, when expressed as a percentage, is the percent measurement noise. We found photobleaching to be insignificant as low and high sorts yielded similar estimates of measurement noise, supporting the assumption of no change in underlying cell fluorescence between measurements. Missorted cells, which have no effective sorting bias, comprised a few percent of sorted cells, and appeared as a minor secondary peak centered on 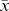, but had little effect on the measurement noise estimates.

### Antibody ELISA

Culture supernatants were removed at each timepoint and stored at −70°C. ELISA was performed as previously described [44]. Specific antibody secretion rates were calculated from endpoint ELISA measurements and cell counts by dividing final antibody yields by log-mean cell number and time elapsed in culture. Standard errors were calculated using an error model accounting for absolute and proportional error sources, along with well-to-well, plate-to-plate, and day-to-day variability in ELISA measurements.

### Data Normalization

Data were normalized for comparison purposes and to correct for day-to-day variability. Fluorescence, in particular, must be standardized for presentation of timecourse data (as units are arbitrary). For sorted subpopulations and subclones, fluorescence at each timepoint was divided by median fluorescence of the parental clone (5H6) at the corresponding timepoint. For the parental clone, fluorescence at each timepoint was normalized to calibration beads, and then divided by the mean bead-normalized fluorescence across all timepoints. Normalization was slightly different for ELISA measurements as these were performed together on retained samples, whereas fluorescence measurements were performed independently at each timepoint. ELISA data was normalized by dividing specific antibody secretion rates for each timepoint by the mean specific antibody secretion rate of the parental clone across all timepoints. Systematic variation in measurement of secretion rate at particular timepoints was offset by subtracting the residual between the parental clone at the corresponding timepoint and its mean. To account for non-expressing cells, both fluorescence and ELISA data were calculated in terms of double-positive cells, which were generally ≥98% of the population in the subclones, but comprised lower percentages in the parental clone (after >100 days in culture). Specifically, median fluorescence was calculated from gated double-positive cells, and antibody secretion rates were divided by the fraction of double-positive cells in the population. Cells expressing only light or heavy chain gave no detectable ELISA signal (data not shown).

### Positive Expression Threshold

The threshold for positive expression was set manually based on the distinct separation of subpopulations on a bivariate dot plot, using untransfected and single-transfected controls as a guide. Cells positive in both EGFP and EYFP fluorescence channels were considered double-positive (‘expressing’) cells. Median fluorescence and the fraction of double-positive cells was not sensitive to precise positioning of the threshold.

### Fitting Relaxation Kinetics

First order exponential decays in **Fig. 3D** and **Fig. 4** were fitted based on three model parameters: initial level (t = 0), steady state level (t→∞), and half-time (t_1/2_). The fits were performed by minimizing mean square error. For fluorescence data, all three parameters were varied. For ELISA data, high scatter relative to signal levels prevented reliable fitting with three parameters. Instead, half-time was set to the value determined for the corresponding fluorescence data, and the remaining two parameters were fit. The assumption of identical kinetics was reasonable based on inspection of the fitted curves (**Fig. 4**). Half-times in **Fig. 3D** were converted to approximate cell generations by assuming a constant cell generation time of 15.3 h, based on population growth data. For the subclones in **Fig. 4**, apparent growth rates varied during the experiment (**Fig. S4**). To address this, cumulative population doublings were calculated from cell yields and inoculation densities at each passage, and curves were independently fit against population doublings to obtain approximate half-times in cell generations.

### Fluorescence Microscopy

Images were captured on an Olympus CKX41 inverted microscope with 50W mercury lamp, U-RFLT50-200 power supply, DM500 dichroic mirror, BA520 IF barrier filter, and BP460-490C excitation filter for EGFP and EYFP fluorescence, using a MicroPublisher 3.3 RTV 10 bit color digital CCD camera and Qcapture Pro 6.0 software (QImaging).

## Supporting Information

Figure S1 Relationship between intracellular reporter fluorescence and cell surface antibody levels.
(0.41 MB PDF)

Figure S2 Steady state expression levels in subclones by multiple methods.
(0.15 MB PDF)

Figure S3 Cell size and doubling time of subclones during long-term culture.
(0.17 MB PDF)

Figure S4 Doubling time and relative expression level of subclones during long-term culture.
(0.15 MB PDF)

Table S1 Mean cell size and relative DNA content of host cells and recombinant clones (at steady state).
(0.02 MB PDF)

Text S1 Supplementary Notes and Methods
(0.02 MB PDF)
