# Supplementary material for: Intraclonal Protein Expression Heterogeneity in Recombinant CHO Cells
Source: PLoS One. 2009 Dec 23;4(12):e8432. doi: 10.1371/journal.pone.0008432 (PMC2793030; doi:10.1371/journal.pone.0008432)
Supplement: Figure S1 — Relationship between intracellular reporter fluorescence and cell surface antibody levels. (0.41 MB PDF) [file pone.0008432.s001.pdf]

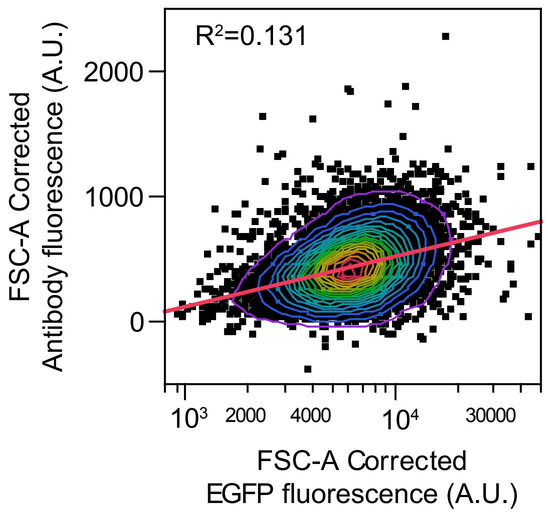

Figure S1. Relationship between intracellular reporter fluorescence and cell surface antibody levels. Higher intracellular EGFP (and EYFP) fluorescence levels were accompanied, on average, by higher cold capture cell surface antibody levels (see Supplementary Methods in **Supporting Information, Text S1**) in individual double-positive cells of parental clone 5H6. This suggests intraclonal variation at the single-cell level is conveyed downstream from transcription to secretion. In order to remove any shared correlation with cell size, EGFP (EYFP) and antibody fluoresence measurements were corrected by FSC-A (Methods) (c.f. R^2=0.186 for uncorrected data, not shown). The correlation between EGFP and EYFP fluorescence was only minimally affected (~1%) by this correction (not shown). Spectral overlap was also fully compensated (Methods). Linear fit (red line) and R^2 are shown. Contours are percentiles (5%). Fluorescence in arbitrary units (A.U.). Note: measurement noise in both assays places an inherent upper limit on R^2. This is more pronounced in single-cell analyses which do not benefit from population averaging. The cold capture method, in particular, was only partially effective in discriminating positive cells from background at the prevailing expression level (signal-to-noise ratio, SNR = $(\mu_{pos} - \mu_{neg})/(\sigma^2_{pos} + \sigma^2_{neg})^{0.5}$ = 0.87, for 5H6 parental clone (*pos*) relative to untransfected control (*neg*), where μ and σ^2 are mean and variance). Intracellular EGFP (and EYFP) measurements were more sensitive and exhibited less overlap with background (SNR = 2.0). Although variation in EGFP (and EYFP) fluorescence was predominantly due to cellular variation (see **Fig. 3**, main text), technical variation probably contributed more substantially to surface antibody measurements due to greater interference from autofluorescence background and possible variations in labeling efficiency.
