# Supplementary material for: Intraclonal Protein Expression Heterogeneity in Recombinant CHO Cells
Source: PLoS One. 2009 Dec 23;4(12):e8432. doi: 10.1371/journal.pone.0008432 (PMC2793030; doi:10.1371/journal.pone.0008432)
Supplement: Figure S2 — Steady state expression levels in subclones by multiple methods. (0.15 MB PDF) [file pone.0008432.s002.pdf]

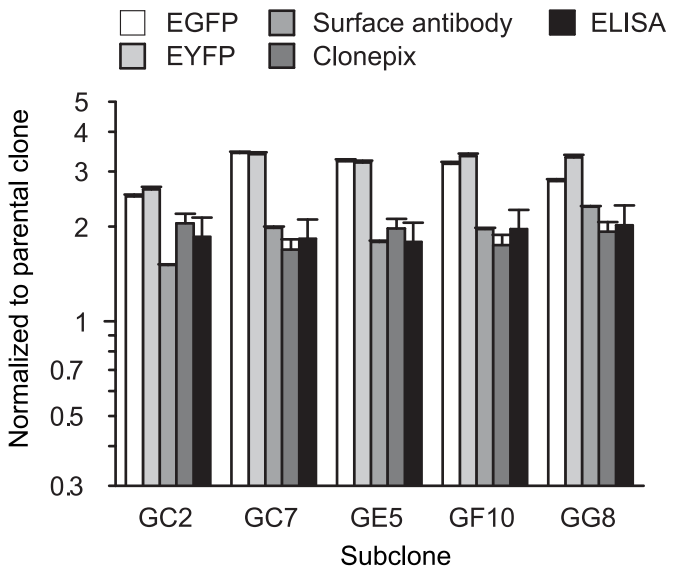

Figure S2. Steady state expression levels in subclones by multiple methods. Using screening assays frequently employed in stable cell line development, we confirmed that the selected subclones possessed steady state expression levels measurably higher than the parental clone. Median intracellular EGFP, EYFP, and cold capture surface antibody fluorescence were determined by flow cytometry. Antibody secretion was measured by ClonePix™ FL at the colony level (see Supplementary Methods in **Supporting Information, Text S1**), and by ELISA (pg/cell-day) at the population level. All analyses were performed after expression had stabilized (>50 days post subcloning). Error bars are standard errors.
