# Supplementary material for: Intraclonal Protein Expression Heterogeneity in Recombinant CHO Cells
Source: PLoS One. 2009 Dec 23;4(12):e8432. doi: 10.1371/journal.pone.0008432 (PMC2793030; doi:10.1371/journal.pone.0008432)
Supplement: Figure S4 — Doubling time and relative expression level of subclones during long-term culture. (0.15 MB PDF) [file pone.0008432.s004.pdf]

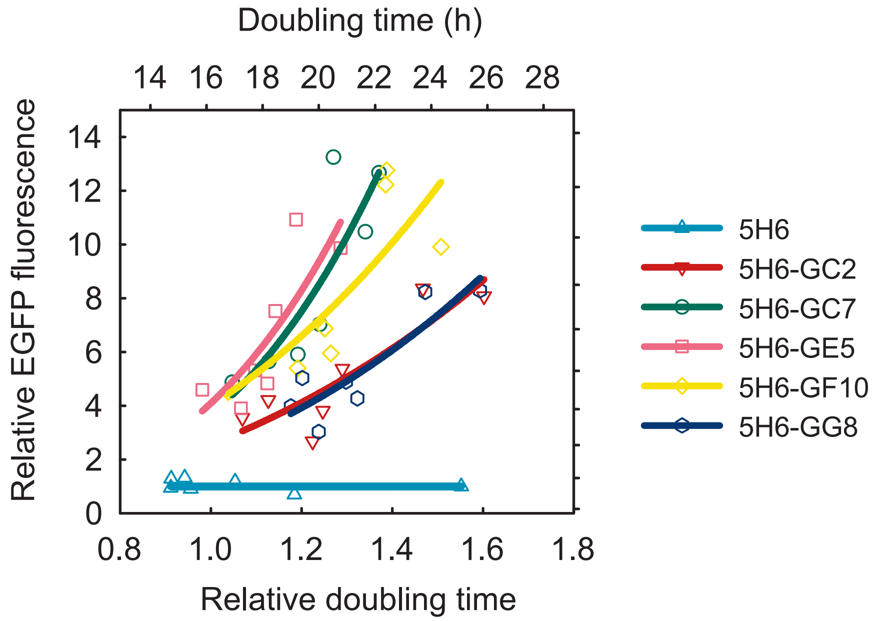

Figure S4. Doubling time and relative expression level of subclones during long-term culture. For each subclone, longer population doubling times correspond, on average, to periods of higher expression. When comparisons are made across subclones the relationship is less clear, at least during the transient decay phase and given the small dataset examined. Normalization and time span are the same as **Fig. S3**. Fitted lines are to aid visual interpretation.
