# Supplementary material for: Intraclonal Protein Expression Heterogeneity in Recombinant CHO Cells
Source: PLoS One. 2009 Dec 23;4(12):e8432. doi: 10.1371/journal.pone.0008432 (PMC2793030; doi:10.1371/journal.pone.0008432)
Supplement: Table S1 — Mean cell size and relative DNA content of host cells and recombinant clones (at steady state). (0.02 MB PDF) [file pone.0008432.s005.pdf]

## Table S1. Mean cell size and relative DNA content of host cells and recombinant clones (at steady state).

| [a]Cell line | [b]Mean cell diameter (μm) | [c]Relative mean cell volume | [d]DNA index |
|---|---|---|---|
| CHO-K1 | 12.9 ± 0.1 | 1 | 1 |
| 5H6 | 12.7 ± 0.1 | 0.96 ± 0.02 | 1.03 ± 0.05 |
| 5H6-GC2 | 13.8 ± 0.1 | 1.24 ± 0.02 | 1.06 ± 0.05 |
| 5H6-GC7 | 13.9 ± 0.1 | 1.26 ± 0.02 | 1.16 ± 0.06 |
| 5H6-GE5 | 13.8 ± 0.1 | 1.23 ± 0.02 | 1.10 ± 0.05 |
| 5H6-GF10 | 14.1 ± 0.1 | 1.32 ± 0.02 | 1.15 ± 0.05 |
| 5H6-GG8 | 15.2 ± 0.1 | 1.66 ± 0.03 | 2.04 ± 0.17 |

[a] Untransfected host cells (CHO-K1), parental clone (5H6), and subclones on Day 57 post-subcloning, after plateau in expression (steady state). [b] Diameter determined by image analysis (Cedex HiRes). Mean from several thousand cells. Assumes circular cross-section. Estimated uncertainty, ± 0.1 μm (standard error). [c] Cell volume relative to CHO-K1, calculated from diameter measurement, assuming spherical cells. Errors propagated. [d] DNA index represents DNA content relative to CHO-K1 (see Supplementary Methods in **Supporting Information, Text S1**). Uncertainty (standard error) includes error estimate for non-linearity correction. Note: altered ploidy is not uncommon in recombinant CHO cell lines [1-3].

## References


1. Derouazi M, Martinet D, Schmutz NB, Flaction R, Wicht M, et al. (2006) Genetic characterization of CHO production host DG44 and derivative recombinant cell lines. Biochemical and Biophysical Research Communications 340: 1069-1077.
2. Jun SC, Kim MS, Hong HJ, Lee GM (2006) Limitations to the development of humanized antibody producing Chinese hamster ovary cells using glutamine synthetase-mediated gene amplification. Biotechnology Progress 22: 770-780.
3. Lattenmayer C, Loeschel M, Steinfellner W, Trummer E, Mueller D, et al. (2006) Identification of transgene integration loci of different highly expressing recombinant CHO cell lines by FISH. Cytotechnology 51: 171-182.
