# Supplementary material for: Intraclonal Protein Expression Heterogeneity in Recombinant CHO Cells
Source: PLoS One. 2009 Dec 23;4(12):e8432. doi: 10.1371/journal.pone.0008432 (PMC2793030; doi:10.1371/journal.pone.0008432)
Supplement: Text S1 — Supplementary Notes and Methods (0.02 MB PDF) [file pone.0008432.s006.pdf]

Pilbrough et al.

Intraclonal Protein Expression Heterogeneity in Recombinant CHO Cells

Supporting Information— Text S1

Supplementary Note 1— Intracellular pH variation

Intracellular pH (pH_i) affects EGFP and EYFP reporter fluorescence [1,2] and this may contribute to noise if pH_i varies from cell to cell. Under physiological conditions, EYFP (pKa ~7.10) is more sensitive to pH_i variation than EGFP (pKa ~6.15) [1], but the observed difference in EGFP and EYFP noise levels was only marginal (EYFP CV higher by 3.0 ± 0.4%, on average). This suggests the contribution of pH_i to fluorescence variation is small relative to other noise sources. Indeed, pH_i variation in CHO cells is reported to be modest (CV 0.04-0.08) [3].

Supplementary Note 2— IRES-specific regulation

Certain theoretical considerations regarding the bicistronic reporter constructs are worthy of mention. The fluorescent reporters and antibody chains are differently regulated at the translational and post-translational level, contributing to potential decoupling of intracellular reporter fluorescence and secretion measurements under some conditions. For example, internal translation initiation from the EMCV IRES is resistant to downregulation of global cap-dependent translation via eIF4E repression in response to environmental stress [4,5]. Similarly, expression from the second cistron may be independently regulated by fluctuations in IRES-specific trans-acting factors such as PTB (polypyrimidine tract-binding protein) [4]. Unlike environmental stress, the EMCV IRES remains sensitive to PERK-mediated eIF2α phosphorylation which downregulates global translation in response to ER stress [4,6]. Thus authentic monitoring of transcription may be compromised by post-translational events, even though the cytoplasmic reporters are not normally subject to other ER stress responses such as ER-associated degradation, which do influence antibody secretion. We are yet to investigate

these factors explicitly, but consider the interpretation presented in the main text to be most consistent with the balance of the evidence under the experimental conditions employed.

Supplementary Methods

Cold Capture Cell Surface Antibody Assay

Cold capture labeling was performed by the method of Brezinsky et al [7]. Briefly, cells were placed immediately on ice, washed with ice cold DPBS and 2% BSA in culture medium, and labeled for 15 min with 10 μg/ml R-phycoerythrin (R-PE)-conjugated $F(ab')_2$ fragment goat anti-human IgG (H+L) (Jackson ImmunoResearch). Additional washes in ice cold DPBS were followed by flow cytometric analysis.

DNA Index Measurement

Samples were spiked with untransfected CHO-K1 cells as an internal control prior to sample processing. DNA content was determined as described for cell cycle analysis (Methods). Peaks and standard errors were determined by curve fitting (Johnson SU distributions). The internal control was discriminated by gating for lack of both EGFP and EYFP fluorescence. Unspiked samples were also analyzed to verify negligible presence of double-negative cells. DNA index was calculated by taking the ratio of modal PI fluorescence of the fitted G0/G1 peak in the sample to that in the internal control. Non-linearity was corrected using a power law model to achieve a geometric mean G2/M to G0/G1 ratio of 2:1 across all samples and internal controls.

ClonePix™ Colony-Level Antibody Secretion Assay

Cells were inoculated at 750 cells/ml in CloneMedia-CHO semi-solid medium (Genetix Ltd.) containing 1.4% methyl cellulose and supplemented with 1% FBS, 5 mM Glutamax®, and selection agents (Methods) in 6-well plates (Genetix Ltd.). CloneDetect® anti-human 649 (Ex) detection antibody (Genetix Ltd.) was added at 100 U/ml. Plates were incubated for 8 days (Methods) and measured under white light, EGFP, EYFP, and Cy5 fluorescence filter sets on a ClonePix™ colony picking robot (Genetix Inc.). Between 500 and 1500 colonies were analyzed for each sample. Colony volume was calculated from the measured colony radius (white light), assuming spherical colony shape. Interior total fluorescence intensity was

divided by colony volume, and log-transformed. Histograms of log-transformed volume-corrected fluorescence were fitted to Johnson SU distributions to extract peak intensities and standard errors. In cases where only one peak could be resolved, background correction was performed by subtracting the sample peak intensity from the peak intensity of the untransfected control plate. In cases where distributions were bimodal, the background corrected signal was taken as the difference between the two peaks. Signals were compensated for spectral overlap using appropriate control plates.

References

1. Llopis J, McCaffery JM, Miyawaki A, Farquhar MG, Tsien RY (1998) Measurement of cytosolic, mitochondrial, and Golgi pH in single living cells with green fluorescent proteins. Proceedings of the National Academy of Sciences of the United States of America 95: 6803-6808.
2. Naciri M, Al-Rubeai M (2006) Non-invasive flow cytometric monitoring of pH(i) in cell culture processes using EGFP. Journal of Immunological Methods 315: 185-190.
3. Lee AH, Tannock IF (1998) Heterogeneity of intracellular pH and of mechanisms that regulate intracellular pH in populations of cultured cells. Cancer Research 58: 1901-1908.
4. Pestova TV, Hellen CUT, Shatsky IN (1996) Canonical eukaryotic initiation factors determine initiation of translation by internal ribosomal entry. Molecular and Cellular Biology 16: 6859-6869.
5. Svitkin YV, Herdy B, Costa-Mattioli M, Gingras AC, Raught B, et al. (2005) Eukaryotic translation initiation factor 4E availability controls the switch between cap-dependent and internal ribosomal entry site-mediated translation. Molecular and Cellular Biology 25: 10556-10565.
6. Spriggs KA, Stoneley M, Bushell M, Willis AE (2008) Re-programming of translation following cell stress allows IRES-mediated translation to predominate. Biology of the Cell 100: 27-38.
7. Brezinsky SCG, Chiang GG, Szilvasi A, Mohan S, Shapiro RI, et al. (2003) A simple method for enriching populations of transfected CHO cells for cells of higher specific productivity. Journal of Immunological Methods 277: 141-155.
