# Supplementary material for: Intraclonal Protein Expression Heterogeneity in Recombinant CHO Cells
Source: PLoS One. 2009 Dec 23;4(12):e8432. doi: 10.1371/journal.pone.0008432 (PMC2793030; doi:10.1371/journal.pone.0008432)
Supplement: Figure S3 — Cell size and doubling time of subclones during long-term culture. (0.17 MB PDF) [file pone.0008432.s003.pdf]

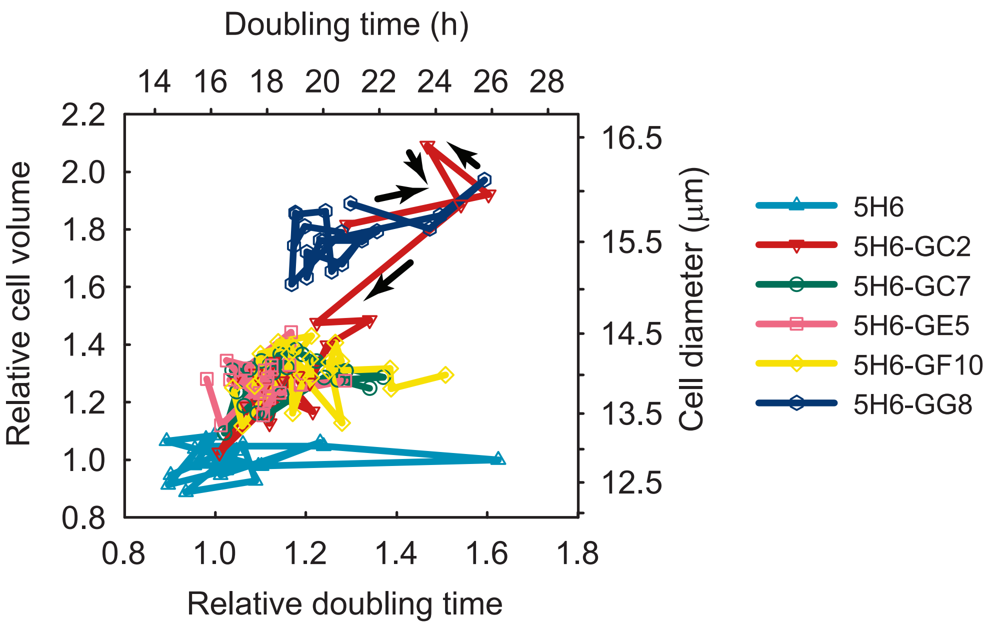

Figure S3. Cell size and doubling time of subclones during long-term culture. The selected subclones have longer apparent doubling times and larger mean cell sizes than the parental clone (5H6), particularly the (DNA) quasi-tetraploid subclone 5H6-GG8. The abrupt shift in cell size occuring in subclone 5H6-GC2 between Day 31 and 39 suggests spontaneous resolution of a similar, though unstable, quasi-tetraploid state. This coincided with cryopreservation of the cell lines, and occurred some time during the first passage post thaw (GC2 mean cell diameter at thaw was 15.6 µm, not shown in figure). The event was reproducible in independent vial thaws. Subclone GG8 was unaffected, remaining quasi-tetraploid. Early quasi-tetraploidy in subclone GC2 could not be confirmed unequivocally as DNA index measurements were only performed after the shift had occurred (**Table S1**). We should further mention that subclones GG8 and GC2 have very similar dynamic expression profiles (**Fig. 4 B, F**, main text) in spite of this event, suggesting that while cell size was significantly impacted, expression was not. Cell size was determined as for **Table S1**. Apparent population doubling times were calculated from endpoint cell counts at each cell passage. Subclones were normalized to the parental clone at each timepoint. The parental clone was normalized to its median over all timepoints. Arrows indicate time progression. Timepoints span 21 to 91 days post subcloning.
